# Comparison between distinct insulin resistance indices in measuring the development of hypertension: The China Health and Nutrition Survey

**DOI:** 10.3389/fcvm.2022.912197

**Published:** 2022-10-06

**Authors:** Yue Yuan, Wei Sun, Xiangqing Kong

**Affiliations:** ^1^Department of Cardiology, The First Affiliated Hospital of Nanjing Medical University, Nanjing, China; ^2^Department of Cardiology, Nanjing Medical University, Nanjing, China

**Keywords:** insulin resistance, hypertension, triglyceride-glucose index, lipid production, lipid

## Abstract

**Aim:**

Our aim was to identify the relationship between several surrogate insulin resistance (IR) indices based on lipid products and the development of hypertension.

**Materials and methods:**

A total of 3,281 participants aged ≥ 18 years enrolled in the China Health and Nutrition Survey from 2009 to 2015 and who were followed up for 6 years were included in the final analysis. Logistic regression was used to analyze the association between different IR indices and incident hypertension.

**Results:**

There were 882 (28.9%) hypertensive participants in 2015. With regard to the homeostasis model assessment of insulin resistance (HOMA-IR) based on insulin level, subjects in the highest quartile of HOMA-IR values were more likely to develop hypertension [RR = 1.58 (1.26–1.98), *P* < 0.001] after being adjusted by sex and age, smoke habits, alcohol consumption, community type, married status, and education years in 2009. Subjects in the highest quartile of the triglyceride-glucose index (TyG) combined with body mass index (BMI) and waist circumference (WC) had more than two times the risk of hypertension after full adjustment compared with individuals in the lowest quartile (both *P* < 0.001), and the trend continued when adjusted for the HOMA-IR. Compared with those in the lowest quartile of TyG-BMI values, females in the highest quartile had a higher risk of developing hypertension than males [2.82 (2.01–3.97) vs. 2.56 (1.80–3.64)] after the full adjustment, and the trend existed independent of IR. Young participants in the highest quartile of the HOMA-IR had significantly higher risks of hypertension compared with subjects in the lowest quartile [1.67 (1.31–2.14), *P* < 0.005], and this trend was not significant in the elderly participants.

**Conclusion:**

The results from our large-scale study elucidate the superiority of the TyG-BMI and TyG-WC compared with the HOMA-IR in the prediction of hypertension, which may be related to lipid deposition. The sex-specific predictive value is distinct for different IR indicators.

## Introduction

Preventing and treating hypertension has become a national priority because of its role in the development of cardiovascular diseases (CVDs) (1, 2). It is predicted that a total of 1.56 billion (1.54–1.58 billion) adults will develop hypertension in 2025 (3). The adverse impact of raised blood pressure on cardiovascular morbidity and mortality is increasing globally, accounting for 10.4 million deaths per year (4, 5). The awareness, treatment, and control rate of hypertension are challenging despite several initiatives in China (6–8). Therefore, it is of great importance to identify predictors of hypertension to improve prevention and management.

Insulin resistance (IR) is the major pathological feature of diabetes and metabolic syndrome, playing a key role in the development of cardiometabolic risks, including hypertension, atherosclerosis, diabetes, artery stiffness, and coronary artery calcification (9, 10). The homeostatic model assessment of insulin resistance (HOMA-IR) based on fasting serum glucose and insulin levels is recognized as a robust marker and is widely used in clinical practice instead of hyperinsulinemic-euglycemic clamp, which is the gold standard test for the measurement of IR (11). The relationship between the HOMA-IR and cardiometabolic disorders has been well-established in both adolescents and adults (12, 13). Recent studies have indicated other surrogate IR indicators based on the combination of lipid indices at different levels, including triglycerides (TGs), high-density lipoprotein cholesterol (HDL-C), body mass index (BMI), and waist circumference (WC), which better applies to epidemiological studies and has been reported to be strongly associated with IR levels (14, 15).

The triglyceride-glucose index (TyG index) calculated from fasting TG and fasting plasma glucose (FPG) levels has been recognized as a reliable surrogate biochemical marker of IR (16–18). Compelling evidence has demonstrated that the TyG plays a principal role in predicting diabetes and coronary artery disease (11, 19) but produces inconsistent results in incident hypertension (20–23). Additionally, the visceral adiposity index (VAI) and lipid accumulation product (LAP) were previously reported to be predictive in the development of diabetes (24). The comparison between the predictive role of the HOMA-IR and its six surrogate IR indices, including the TyG, TG/ HDL-C, VAI, LAP, TyG-BMI, and TyG-WC, in incident hypertension has never been conducted before.

The aims of the present study are (1) to indicate the predictive role of IR by the HOMA-IR in the development of hypertension through a longitudinal open cohort study, (2) to compare the effect of the TyG index and the other five surrogate IR indices on later incident hypertension, and (3) to define sex- and age-specific relationships between IR indices and hypertension. Examining whether the abovementioned indices are superior to the HOMA-IR has important implications for reducing the burden of hypertension *via* longitudinal analyses of a large prospective cohort study in China.

## Materials and methods

### Study design

**Figure 1:**
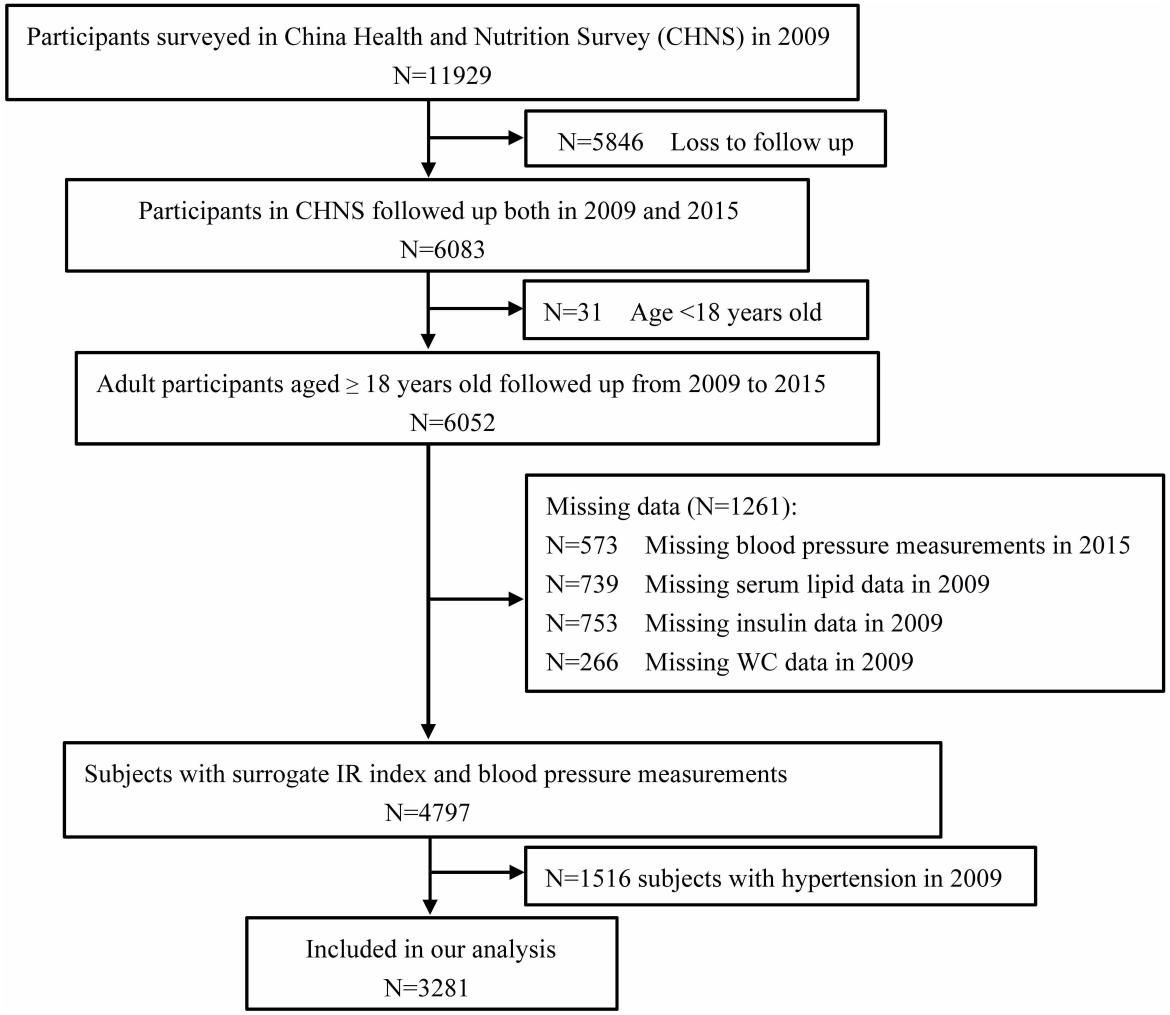
Flowchart of the study participants from the China Health and Nutrition Survey (*n* = 3,281).

The China Health and Nutrition Survey (CHNS) is an ongoing observational open cohort study for monitoring and understanding socioeconomic and health changes in China. Detailed cohort enrollment and information have been previously described (25, 26). Cohort study materials and acknowledgment are available at the website http://www.cpc.unc.edu/projects/china. The examination was conducted in 1989, 1991, 1993, 1997, 2000, 2004, 2006, 2009, and 2015. Trained staff collected data on demographic characteristics and basic anthropometrics measurements during each follow-up of participants from nine provinces, including Liaoning, Heilongjiang, Jiangsu, Shandong, Henan, Hubei, Hunan, Guangxi, and Guizhou. Blood samples were first collected in 2009. In this study, we analyzed the data from the CHNS between 2009 and 2015. Participants who were missing information on biomarkers from blood samples in 2009 and anthropometric measurements, including WC measurements, and individuals aged < 18 years were excluded from the present study. We further excluded 622 subjects with hypertension history and antihypertensive treatment by questionnaires and 894 subjects whose average systolic blood pressure (SBP) measured in 2009 was ≥ 140 mmHg or diastolic blood pressure (DBP) was ≥ 90 mmHg. The flow chart of the target population of this current study from the CHNS cohort study is shown in Figure 1. Finally, 3,281 participants (1,413 males and 1,868 females) who were followed up for 6 years were included in this analysis. Written informed consent was obtained from each adult participant. The CHNS study was approved by the institutional review committees of the National Institute of Nutrition and Food Safety, the Chinese Center for Disease Control and Prevention, the University of North Carolina at Chapel Hill, and the China-Japan Friendship Hospital, Ministry of Health. The protocols were in accordance with relevant guidelines and regulations.

### Examination methods

The demographic characteristics of the subjects were collected by standard questionnaires and included age, sex, community type (rural and urban), years of education, material status, smoking habits, alcohol consumption, and medical history of hypertension, diabetes, stroke, and medical treatment. WC was measured at a point midway between the lowest rib and the iliac crest in a horizontal plane using a non-elastic tape, and hip circumference was measured at the level of the maximum extension of the buttocks posteriorly in a horizontal plane with the participants wearing light clothes and arms open sideways. Height was measured to the nearest 0.1 cm without shoes using a portable stadiometer, and weight was measured to the nearest 0.1 kg while participants were wearing lightweight clothing. BMI (kg/m^2^) was calculated as weight (kilograms) divided by height (meters) squared. BP was measured three times *via* standard mercury sphygmomanometers in the seated position after at least 10 min of rest. Phase I and V Korotkoff sounds were recorded as SBP and DBP. Mean arterial pressure (MAP, mmHg) was calculated by the formula MAP = 1/3 SBP+ 2/3 DBP.

Biochemical assays of the blood samples (12 mL) were collected after subjects fasted overnight for at least 8 h according to the standard protocol and guidelines. Trained staff transferred the collected blood samples to the local hospital for further testing. A detailed biochemical assessment is available at the website: https://www.cpc.unc.edu/projects/china/data/datasets/biomarker-data. All biochemical assessments were conducted in a national central laboratory in Beijing (Medical laboratory accreditation certificate: ISO 15189:2007) (27). Hematologic analysis was performed by Beckman Coulter LH750, USA. FPG was measured by the GOD-PAP method (Randox Laboratories Ltd, UK). Apolipoprotein A (ApoA) and apolipoprotein B (ApoB) were measured by immunoturbidimetric methods (Randox Laboratories Ltd, UK). Fasting insulin concentration was measured by the radioimmunology assay (Gamma counter XH-6020, Beijing, China). Other biochemical indicators, including TG, total cholesterol (TC), low-density lipoprotein cholesterol (LDL-C), HDL-C, urea, serum uric acid, serum creatinine, total protein, albumin, and alanine aminotransferase, were measured by a Hitachi 7600 automated analyzer (Hitachi Inc., Tokyo, Japan).

### Definitions

In the present study, subjects with hypertension were defined as those with SBP ≥ 140 or DBP ≥ 90 mmHg or who were receiving antihypertension treatment. Subjects with FPG ≥ 7.0 mmol/L or who were receiving oral hypoglycemic drugs and insulin were considered to have diabetes mellitus (28). The assessment of IR was as follows:

The HOMA-IR was calculated by the formula: HOMA-IR = fasting insulin (microinternational units per milliliter) × FPG (millimoles per liter)/22.5 (15).

The homeostasis model assessment of β-cell function (HOMA- β) was calculated as 20 × fasting insulin (μU/mL)/ [FPG (mmol/L) – 3.5] (29, 30).

Surrogate IR indices were calculated according to previously published formulas:

TyG index as Ln [TG (mg/dL) × FPG (mg/dL)/2] (15);

TG/HDL-C as the ratio between serum TGs and HDL-C (31);

VAI (males) = WC (cm) /(39.68 + 1.88 × BMI) × [TG (mmol/L)/ 1.03] × [1.31/HDL-C (mmol/L)] (32);

VAI (females) = WC (cm) /(36.58 + 1.89 × BMI) × [TG (mmol/L)/0.81] × [1.52/HDL-C (mmol/L)] (33);

Lipid accumulation product (LAP) for males: LAP = [WC (cm) − 65] × [TG (mmol/L)] and [WC (cm) − 58] × [TG (mmol/L)] for females (34);

TyG-BMI = TyG index × BMI;

TyG-WC = TyG index × WC (16).

### Statistical analysis

All analyses were conducted in SPSS version 25.0 for Windows (SPSS Inc., Chicago, IL, USA). Demographic characteristics are shown according to different BP groups and IR index categories. Continuous variables are shown as the mean ± SD if normally distributed or median (quartile 1, quartile 3) if non-normally distributed. Categorical variables are expressed as numbers and percentages of subjects. Statistical ANOVA was performed by one-way ANOVA when normally distributed; otherwise, the Mann–Whitney *U*-test was used. Differences between groups of categorical variables were compared with χ^2^-tests. A logistic model was conducted to identify the relationship between the HOMA-IR and the surrogate IR index and the development of incident hypertension. Three models were set to adjust the risk ratios (RRs), and 95% confidence intervals (CIs) were conducted to analyze the relationship between IR indices and incident hypertension in this study. Model 1 was adjusted for sex and age in 2009; Model 2 included Model 1 + smoking habits, alcohol consumption, community type, marital status, and years of education; and Model 3 included Model 2 + WC, urea, serum uric acid, serum creatinine, HDL-C, LDL-C, TC, LDL-C, white blood cell count, red blood cell count, platelet count, hemoglobin A1c, hemoglobin, total protein, albumin, alanine aminotransferase, ApoA, and ApoB. A two-tailed *p* < 0.05 was considered to be statistically significant.

## Results

### Clinical characteristics of the participants included in the longitudinal analysis

A comparison of characteristics and cardiovascular risks between subjects with hypertension and those without hypertension in 2015 is presented in Table 1. The median age of the participants was 46.00 (38.00–56.00) years in individuals with normal BP and 53.00 (45.00–61.00) years in hypertensive individuals. Males were more likely to suffer from hypertension during the follow-up. Compared with subjects with normal BP, hypertensive subjects had significantly higher BMI, hip circumference, BP, levels of urea, serum uric acid, creatinine, LDL-C, TC, TG, red blood cell count, hemoglobin A1c, hemoglobin, total protein, FPG, alanine aminotransferase, and ApoB in 2009 (all *P-*values < 0.05). Regarding the IR index, the levels of the HOMA-IR, TyG, TG/HDL-C, and TyG-BMI were higher in those with hypertension than in those with normal BP (all *P-*values < 0.05). Because WC was sex-specific, we performed the comparison in WC, VAI, LAP, and TyG-WC between subjects with normal blood pressure and those with hypertension (Supplementary Table 1), and found that compared with subjects with normal BP, males with hypertension had significantly higher WC, VAI, LAP, and TyG-WC than males with normal blood pressure, and the trend was similar in females. In addition, subjects with higher BP were associated with high rates of smoking, drinking, and diabetes. Those with normal BP were more likely to be married, less educated, and live in urban areas (all *P-*values < 0.05).

**Table 1:** Comparison of characteristics and cardiovascular risks between target population with normal blood pressure and subjects with incident hypertension.

**Parameter**	**Subjects with normal blood pressure** **(*n* = 2,399)**	**Subjects with hypertension (*n* = 882)**	***P*-value**
Gender (%)			0.003
Male	996 (41.5)	417 (47.3)	
Parameter in 2009			
Age, yr	46 (38–56)	53 (45–61)	< 0.001
Living area (%)			< 0.001
Urban	736 (30.7)	204 (23.1)	
Rural	1663 (69.3)	678 (76.9)	
Marital status (%)			0.263
Married	2153 (89.7)	796 (90.2)	
Divorced	33 (1.4)	6 (0.7)	
Unmarried or other	213 (8.9)	80 (9.1)	
Education year, yr	7.55 ± 4.40	6.45 ± 4.64	< 0.001
Smoking (%)	696 (29.0)	284 (32.2)	0.077
Drinking (%)	752 (31.0)	327 (37.1)	0.001
Diabetes (%)	24 (1.0)	19 (2.2)	< 0.001
BMI, kg/m^2^	22.6 ± 3.1	23.8 ± 3.3	< 0.001
WC, cm	80 (73–87)	84 (77–91)	< 0.001
Hip, cm	93 (88–97)	95 (90–100)	< 0.001
SBP, mm Hg	116 (108–121)	121 (116–129)	< 0.001
DBP, mm Hg	77 (70–80)	80 (89–97)	< 0.001
MAP, mm Hg	90 (83–94)	93 (89–97)	< 0.001
Urea, mmol/L	5.35 ± 1.50	5.54 ± 1.44	< 0.001
Serum uric acid, μmol/L	277 (225–339)	294 (241–355)	< 0.001
Serum creatinine, μmol/L	83 (74–94)	85 (76–95)	0.002
HDL-C, mmol/L	1.46 ± 0.45	1.44 ± 0.41	0.319
LDL-C, mmol/L	2.87 ± 0.85	3.08 ± 1.03	< 0.001
TC, mmol/L	4.74 ± 0.93	4.97 ± 0.99	< 0.001
Triglycerides, mmol/L	1.52 ± 1.28	1.70 ± 1.43	< 0.001
Insulin, IU/mL	9.77 (6.99–13.98)	10.16 (7.10–14.69)	0.111
White blood cell count, 10^9^/L	6.13 ± 1.88	6.19 ± 1.69	0.196
Red blood cell count, 10^12^/L	4.65 ± 0.68	4.75 ± 0.68	< 0.001
Platelet count, 10^9^/L	213.85 ± 67.39	213.20 ± 71.00	0.871
Hemoglobin A1c, %	5.48 ± 0.68	5.69 ± 0.95	< 0.001
Hemoglobin, g/L	139.13 ± 20.48	142.47 ± 20.74	< 0.001
Total protein, g/L	77 (73–80)	77 (74–81)	0.028
Albumin, g/L	47 (45–49)	47 (45–50)	0.052
Fasting plasma glucose, mmol/L	5.19 ± 1.14	5.47 ± 1.45	< 0.001
Alanine Aminotransferase, U/L	18 (13–25)	19 (14–27)	< 0.001
Apolipoprotein A, g/L	111 (96–129)	109 (95–130)	0.838
Apolipoprotein B, g/L	84 (70–102)	91 (76–109.25)	< 0.001
HOMA-IR	2.18 (1.52–3.20)	2.38 (1.62–3.57)	0.001
HOMA-β	132.94 (89.83–199.22)	119.24 (80.70–186.86)	< 0.001
TyG	8.39 (7.99–8.85)	8.56 (8.15–9.03)	< 0.001
TG/HDL-C	0.81 (0.51–1.36)	0.92 (0.57–1.52)	< 0.001
VAI	1.29 (0.79–2.25)	1.48 (0.90–2.47)	< 0.001
LAP	21.90 (10.75–39.20)	28.65 (15.80–48.39)	< 0.001
TyG–BMI	188.17 (166.18–213.04)	202.00 (177.58–229.14)	< 0.001
TyG–WC	668.64 (595.81–752.33)	715.72 (643.90–800.79)	< 0.001
**Parameter in 2015**			
SBP in 2015, mm Hg	120 (111–127)	143 (137–151)	< 0.001
DBP in 2015, mm Hg	79 (71–81)	87 (80–94)	< 0.001
MAP in 2015, mm Hg	92 (86–96)	107 (102–113)	< 0.001
BMI in 2015, kg/m^2^	23.2 (21.2–25.4)	23.8 (21.5–26.1)	< 0.001
WC in 2015, cm	82 (75–89)	87 (80–94)	< 0.001
Hip in 2015, cm	94 (89–99)	96 (91–101)	< 0.001

BMI, body mass index; WC, waist circumference; SBP, systolic blood pressure; DBP, diastolic blood pressure; MAP, mean arterial pressure; HDL-C, high-density lipoprotein cholesterol; LDL-C, low-density lipoprotein cholesterol; TC, total cholesterol; HOMA-IR, homeostasis model assessment of insulin resistance; HOMA-β, homeostasis model assessment of β-cell function; TyG, triglyceride and glucose; VAI, visceral adiposity index; LAP, lipid accumulation product.

Continuous variables are shown as mean ± SD if normally distributed or median (quartile 1, quartile 3) if non-normally distributed. Categorical variables are expressed as numbers and percentages of subjects.

Education year, BMI, urea, HDL-C, LDL-C, TC, triglycerides, white blood cell count, red blood cell count, platelet count, hemoglobin A1c, hemoglobin, and fasting plasma glucose in 2009 were normally distributed.

### Prediction of hypertension by distinct IR indices

There were 882 (26.9%) hypertensive participants among the 3,281 participants in 2015. Table 2 shows the predictive role of different IR indices in the development of hypertension. With regard to the HOMA-IR based on insulin level, subjects in the highest quartile of the HOMA-IR levels were more likely to develop hypertension [RR = 1.58 (1.26–1.98), *P* < 0.001] after adjustment for sex and age, smoke habits, alcohol consumption, community type, married status, and education years in 2009, but this was not significant after the full adjustment. The predictive role of the surrogate IR index (LAP, TyG-BMI, and TyG-WC) in incident hypertension was significant after the full adjustment model. Among these IR indices, subjects in the highest quartile of the TyG-WC and TyG-BMI had more than two times the risk of hypertension after full adjustment compared with individuals in the lowest quartile (both *P* < 0.001). However, the trend was not significant in the prediction of hypertension according to the HOMA-β categories.

**Table 2:** Risk ratios and 95% confidence intervals of the association of IR categories and its surrogate index with incident hypertension.

**Categories**	**Incident hypertension (%)**	**Model 1**			**Model 2**			**Model 3**		
		**RR**	**95% CI**	***P-*value**	**RR**	**95% CI**	***P-*value**	**RR**	**95% CI**	***P-*value**
**HOMA-IR categories**										
Quartile 1	198 (24.1)	1	-	-	1	-	-	1	-	-
Quartile 2	204 (25.0)	1.10	0.88–1.39	0.400	1.11	0.88–1.41	0.369	0.98	0.77–1.25	0.870
Quartile 3	221 (26.8)	1.32	1.05–1.67	**0.018**	1.36	1.07–1.72	**0.011**	1.10	0.85–1.42	0.457
Quartile 4	259 (31.6)	1.53	1.22–1.91	**< 0.001**	1.58	1.26–1.98	**< 0.001**	1.19	0.92–1.55	0.188
**HOMA-β** **categories**										
Quartile 1	255 (31.1)	1	-	-	1	-	-	1	-	-
Quartile 2	228 (27.8)	0.93	0.75–1.16	0.528	0.95	0.76–1.19	0.659	0.97	0.77–1.22	0.788
Quartile 3	201 (24.5)	0.86	0.69–1.08	0.187	0.87	0.69–1.09	0.221	0.85	0.67–1.08	0.180
Quartile 4	198 (24.1)	0.88	0.70–1.11	0.278	0.93	0.74–1.17	0.537	0.92	0.72–1.17	0.489
**TyG categories**										
Quartile 1	161 (19.8)	1	-	-	1	-	-	1	-	-
Quartile 2	207 (25.2)	1.29	1.02–1.64	**0.036**	1.32	1.03–1.68	**< 0.001**	1.15	0.88–1.49	0.310
Quartile 3	250 (30.2)	1.66	1.32–2.10	**< 0.001**	1.67	1.32–2.12	**< 0.001**	1.35	1.01–1.80	**0.041**
Quartile 4	264 (32.4)	1.74	1.38–2.20	**< 0.001**	1.76	1.39–2.23	**< 0.001**	1.43	1.00–2.06	**0.050**
**TG/HDL-C categories**										
Quartile 1	185 (22.6)	1	-	-	1	-	-	1	-	-
Quartile 2	202 (24.8)	1.12	0.88–1.41	0.355	1.13	0.89–1.43	0.310	0.10	0.75–1.28	0.901
Quartile 3	252 (30.4)	1.48	1.18–1.86	**0.001**	1.52	1.21–1.92	**< 0.001**	1.62	1.22–2.16	**0.001**
Quartile 4	243 (29.7)	1.40	1.12–1.77	**0.004**	1.44	1.15–1.82	**0.002**	1.84	1.31–2.95	**< 0.001**
**VAI categories**										
Quartile 1	183 (22.4)	1	-	-	1	-	-	1	-	-
Quartile 2	211 (25.6)	1.27	1.01–1.61	**0.044**	1.30	1.02–1.64	**0.032**	1.18	0.90–1.53	0.231
Quartile 3	247 (30.0)	1.65	1.30–2.10	**< 0.001**	1.69	1.32–2.14	**0.036**	1.62	1.22–2.16	**0.001**
Quartile 4	241 (29.4)	1.53	1.21–1.94	**< 0.001**	1.55	1.22–1.96	**< 0.001**	1.05	0.73–1.50	0.811
**LAP categories**										
Quartile 1	148 (18.0)	1	-	-	1	-	-	1	-	-
Quartile 2	208 (25.4)	1.53	1.20–1.96	**0.001**	1.53	1.19–1.96	**0.001**	1.39	1.06–1.81	**0.016**
Quartile 3	253 (30.8)	2.02	1.59–2.57	**< 0.001**	2.03	1.59–2.60	**< 0.001**	1.62	1.22–2.16	**0.001**
Quartile 4	273 (33.3)	2.2	1.67–2.69	**< 0.001**	2.13	1.68–2.72	**< 0.001**	1.84	1.31–2.59	**< 0.001**
**TyG-BMI categories**										
Quartile 1	142 (17.3)	1	-	-	1	-	-	1	-	-
Quartile 2	193 (23.5)	1.49	1.16–1.92	**0.002**	1.57	1.22–2.03	**0.001**	1.47	1.13–1.92	**0.003**
Quartile 3	245 (29.8)	2.08	1.63–2.65	**< 0.001**	2.12	1.66–2.72	**< 0.001**	1.94	1.46–2.57	**< 0.001**
Quartile 4	302 (36.8)	2.81	2.22–3.55	**< 0.001**	2.82	2.22–3.59	**< 0.001**	2.62	1.92–3.59	**< 0.001**
**TyG-WC categories**										
Quartile 1	124 (15.1)	1	-	-	1	-	-	1	-	-
Quartile 2	209 (25.4)	1.78	1.38–2.29	**< 0.001**	1.76	1.36–2.27	**< 0.001**	1.69	1.29–2.23	**< 0.001**
Quartile 3	244 (29.7)	2.09	1.63–2.69	**< 0.001**	2.10	1.63–2.70	**< 0.001**	1.88	1.40–2.53	**< 0.001**
Quartile 4	305 (37.2)	2.86	2.24–3.64	**< 0.001**	2.85	2.23–3.64	**< 0.001**	2.44	1.74–3.43	**< 0.001**

Model 1: adjusted for sex and age in 2009.

Model 2: model 1+ smoke habits, alcohol consumption, community type, married status and education years.

Model 3: model 2+ urea, serum uric acid, serum creatinine, low-density lipoprotein cholesterol, total cholesterol, white blood cell count, red blood cell count, platelet count, hemoglobin A1c, hemoglobin, total protein, albumin, alanine aminotransferase, apolipoprotein A, and apolipoprotein B.

HOMA-IR, homeostasis model assessment of insulin resistance; HOMA-β, homeostasis model assessment of β-cell function; TyG, triglyceride and glucose; VAI, visceral adiposity index; LAP, lipid accumulation product; BMI, body mass index.

Significant values were in bold.

### Sex-specific predictive role in hypertension by IR index

We further analyzed the relationship between the IR index and the presence of hypertension by sex, which is shown in Table 3. Compared with those in the lowest quartile of the HOMA-IR, females in the highest quartile had higher risks of developing hypertension than males [1.71 (1.24–2.36) vs. 1.40 (1.01–1.95)] after adjustment for age, smoking habits, alcohol consumption, community type, marital status, and years of education. Similar trends existed in predicting hypertension between males and females according to the LAP, TyG-BMI, and TyG-WC indices. Females in the highest quartile of the TyG-WC had nearly three times the risk of hypertension [adjusted RR = 2.91 (2.05–4.14), *P* < 0.001] compared to those in the lowest quartile, while the corresponding RR was 2.53 (1.76–3.62) in males. There was no significant role of HOMA-β categories in predicting incident hypertension.

**Table 3:** Risk ratios and 95% confidence intervals of the association of IR categories and its surrogate index with incident hypertension by gender.

**Incident hypertension**	**Male**						**Female**					
**Groups**	**Model 1**			**Model 2**			**Model 1**			**Model 2**		
	**RR**	**95% CI**	***P-*value**	**RR**	**95% CI**	***P-*value**	**RR**	**95% CI**	***P-*value**	**RR**	**95% CI**	***P-*value**
**HOMA-IR categories**												
Quartile 1	1	—	—	1	—	—	1	—	—	1	—	—
Quartile 2	0.89	0.64–1.25	0.507	0.91	0.65–1.28	0.583	1.33	0.96–1.84	0.087	1.34	0.96–1.86	0.084
Quartile 3	1.10	0.80–1.54	0.537	1.18	0.84–1.64	0.342	1.54	1.10–2.16	**0.011**	1.54	1.10–2.17	**0.013**
Quartile 4	1.34	0.97–1.84	0.072	1.40	1.01–1.95	**0.042**	1.68	1.22–2.31	**0.001**	1.71	1.24–2.36	**0.001**
**HOMA-β** **categories**												
Quartile 1	1	—	—	1	—	—	1	—	—	1	—	—
Quartile 2	0.83	0.60–1.14	0.242	0.87	0.63–1.20	0.390	1.03	0.76–1.40	0.845	1.05	0.77–1.44	0.748
Quartile 3	0.91	0.65–1.26	0.556	0.95	0.68–1.32	0.766	0.83	0.60–1.13	0.230	0.82	0.60–1.12	0.213
Quartile 4	0.82	0.60–1.14	0.239	0.90	0.64–1.25	0.512	0.95	0.69–1.31	0.762	0.97	0.70–1.34	0.852
**TyG categories**												
Quartile 1	1	—	—	1	—	—	1	—	—	1	—	—
Quartile 2	1.33	1.12–1.58	**0.001**	1.22	0.85–1.76	0.278	1.19	1.01–1.40	0.318	1.34	0.96–1.87	0.087
Quartile 3	1.77	1.26–2.49	**0.001**	1.79	1.27–2.53	**0.001**	1.41	1.02–1.95	**0.038**	1.42	1.02–1.98	**0.036**
Quartile 4	1.76	1.26–2.45	**0.001**	1.77	1.26–2.49	**0.001**	1.57	1.12–2.20	**0.009**	1.62	1.15–2.27	**0.006**
**TG/HDL-C categories**												
Quartile 1	1	—	—	1	—	—	1	—	—	1	—	—
Quartile 2	0.89	0.62–1.98	0.503	0.91	0.63–1.32	0.626	1.27	0.93–1.74	0.130	1.24	0.91–1.70	0.176
Quartile 3	1.41	1.01–1.98	**0.046**	1.51	1.07–2.14	**0.019**	1.41	1.03–1.93	**0.034**	1.40	1.01–1.92	**0.042**
Quartile 4	1.32	0.95–1.84	0.103	1.38	0.98–1.95	0.064	1.31	0.95–1.82	0.101	1.32	0.95–1.83	0.101
**VAI categories**												
Quartile 1	1	—	—	1	—	—	1	—	—	1	—	—
Quartile 2	1.01	0.73–1.39	0.961	1.04	0.75–1.44	0.832	1.61	1.13–2.31	**0.009**	1.54	1.07–2.22	**0.019**
Quartile 3	1.60	1.12–2.20	**0.004**	1.66	1.20–2.30	**0.002**	1.56	1.09–2.24	**0.015**	1.54	1.07–2.21	**0.020**
Quartile 4	1.26	0.90–1.75	0.177	1.96	0.89–1.75	0.196	1.65	1.16–2.35	**0.006**	2.65	1.65–2.36	**0.007**
**LAP categories**												
Quartile 1	1	—	—	1	—	—	1	—	—	1	—	—
Quartile 2	1.52	1.10–2.12	**0.012**	1.54	1.10–2.16	**0.012**	1.50	1.03–2.18	**0.034**	1.46	1.00–2.13	0.051
Quartile 3	1.93	1.39–2.67	**< 0.001**	1.89	1.35–2.64	**< 0.001**	1.96	1.36–2.82	**< 0.001**	1.97	1.36–2.84	**< 0.001**
Quartile 4	1.84	1.33–2.54	**< 0.001**	1.79	1.28–2.51	**0.001**	2.28	1.58–3.29	**< 0.001**	2.28	1.57–3.30	**< 0.001**
**TyG-BMI categories**												
Quartile 1	1	—	—	1	—	—	1	—	—	1	—	—
Quartile 2	1.35	0.94–1.94	0.100	1.41	0.97–2.03	0.070	1.61	1.13–2.28	**0.008**	1.69	1.18–2.41	**0.004**
Quartile 3	2.03	1.44–2.86	**< 0.001**	1.98	1.39–2.80	**< 0.001**	2.02	1.43–2.86	**< 0.001**	2.13	1.49–3.03	**< 0.001**
Quartile 4	2.69	1.92–3.76	**< 0.001**	2.56	1.80–3.64	**< 0.001**	2.79	1.99–3.90	**< 0.001**	2.82	2.01–3.97	**< 0.001**
**TyG-WC categories**												
Quartile 1	1	—	—	1	—	—	1	—	—	1	—	—
Quartile 2	1.74	1.19–2.54	**0.04**	1.70	1.21–2.41	**0.002**	1.73	1.23–2.44	**0.002**	1.68	1.15–2.48	**0.008**
Quartile 3	1.88	1.29–2.73	**0.001**	1.81	1.23–2.65	**0.002**	2.25	1.60–3.15	**< 0.001**	2.21	1.56–3.12	**< 0.001**
Quartile 4	2.63	1.85–3.74	**< 0.001**	2.53	1.76–3.62	**< 0.001**	2.81	1.99–3.98	**< 0.001**	2.91	2.05–4.14	**< 0.001**

Model 1: adjusted for age in 2009.

Model 2: model 1+ smoke habits, alcohol consumption, community type, married status and education years.

HOMA-IR, homeostasis model assessment of insulin resistance; HOMA-β, homeostasis model assessment of β-cell function; TyG, triglyceride and glucose; VAI, visceral adiposity index; LAP, lipid accumulation product; BMI, body mass index.

Significant values were in bold.

### Age-specific predictive role in hypertension by IR index

Supplementary Table 2 showed the comparison of characteristics and cardiovascular risks between the young participant group (18 ≤ age ≤ 64 years) and the elderly participant group (age ≥ 65 years). Elderly participants had significantly higher levels of uric acid, creatinine, and LDL-C (*P* < 0.005) than young participants. Table 4 presents further analysis of the risks of hypertension for the IR index by different age groups. Young participants in the highest quartile of the HOMA-IR seemed to have significantly higher risks of hypertension compared with the subjects in the lowest quartile [1.67 (1.31–2.14), *P* < 0.005], and this trend was not significant in the elderly participants. With regard to the VAI, LAP, TyG-BMI, and the TyG-WC index, young participants in the highest quartile seemed to have slightly higher risks of hypertension compared with the subjects in the lowest quartile. Contrary to this trend, the risk of hypertension was higher in elderly individuals than in young individuals when compared in quartile 4 and quartile 1 of TyG [2.16 (1.09–4.26) vs. 1.88 (1.46–2.41)].

**Table 4:** Risk ratios and 95% confidence intervals of the association of IR categories and its surrogate index with incident hypertension by age periods.

**Incident hypertension**	**Yong participants**						**Elderly participants**					
	**(18** ≤ **age** ≤ **64 years old)**						**(age** ≥**65 years old)**					
**Groups**	**Model 1**			**Model 2**			**Model 1**			**Model 2**		
	**RR**	**95% CI**	***P-*value**	**RR**	**95% CI**	***P-*value**	**RR**	**95% CI**	***P-*value**	**RR**	**95% CI**	***P-*value**
**HOMA-IR categories**												
Quartile 1	1	—	—	1	—	—	1	—	—	1	—	—
Quartile 2	1.12	0.88–1.44	0.355	1.17	0.91–1.50	0.234	0.91	0.51–1.63	0.747	0.88	0.48–1.63	0.688
Quartile 3	1.18	0.92–1.51	**0.188**	1.26	0.98–1.62	0.069	1.66	0.89–3.09	0.112	1.61	0.85–3.05	0.141
Quartile 4	1.56	1.56–1.99	**< 0.001**	1.67	1.31–2.14	**< 0.001**	1.17	0.67–2.04	0.583	1.13	0.64–2.01	0.677
**HOMA-β** **categories**												
Quartile 1	1	—	—	1	—	—	1	—	—	1	—	—
Quartile 2	0.83	0.66–1.06	0.131	0.86	0.68–1.10	0.232	1.22	0.72–2.06	0.468	1.24	0.72–2.14	0.430
Quartile 3	0.79	0.62–1.00	**0.047**	0.81	0.64–1.04	0.094	0.83	0.83–1.52	0.540	0.76	0.40–1.45	0.406
Quartile 4	0.77	0.61–0.98	**0.033**	0.84	0.66–1.08	0.170	0.65	0.65–1.22	0.180	0.70	0.37–1.33	0.271
**TyG categories**												
Quartile 1	1	—	—	1	—	—	1	—	—	1	—	—
Quartile 2	1.33	1.03–1.72	**0.030**	1.37	1.06–1.78	**0.017**	1.23	0.66–2.30	0.520	1.13	0.59–2.16	0.709
Quartile 3	1.81	1.41–2.31	**< 0.001**	1.28	1.39–2.28	**< 0.001**	1.31	0.68–2.52	0.426	1.49	0.74–2.98	0.263
Quartile 4	1.86	1.45–2.38	**< 0.001**	1.88	1.46–2.41	**< 0.001**	1.87	0.98–3.55	0.057	2.16	1.09–4.26	**0.027**
**TG/HDL-C categories**												
Quartile 1	1	—	—	1	—	—	1	—	—	1	—	—
Quartile 2	1.17	0.91–1.51	0.217	1.19	0.92–1.53	0.188	0.88	0.49–1.57	0.659	0.92	0.51–1.67	0.790
Quartile 3	1.53	1.20–1.95	**0.001**	1.56	1.22–2.00	**< 0.001**	1.21	0.67–2.20	0.523	1.19	0.64–2.20	0.580
Quartile 4	1.44	1.13–1.85	**0.004**	1.49	1.16–1.91	**0.002**	1.14	0.62–2.08	0.673	1.25	1.25–2.39	0.503
**VAI categories**												
Quartile 1	1	—	—	1	—	—	1	—	—	1	—	—
Quartile 2	1.38	1.07–1.77	**0.013**	1.41	1.10–1.82	**0.008**	0.77	0.41–1.44	0.416	0.81	0.43–1.53	0.514
Quartile 3	1.79	1.39–2.30	**< 0.001**	1.81	1.40–2.34	**< 0.001**	1.22	0.66–2.25	0.519	1.26	0.67–2.36	0.480
Quartile 4	1.64	1.64–2.10	**< 0.001**	1.67	1.30–2.15	**< 0.001**	1.07	1.07–2.04	0.838	1.09	0.56–2.11	0.810
**LAP categories**												
Quartile 1	1	—	—	1	—	—	1	—	—	1	—	—
Quartile 2	1.56	1.20–2.03	**0.001**	1.54	1.18–2.01	**0.002**	2.24	1.19–4.19	**0.012**	2.21	1.17–4.18	**0.014**
Quartile 3	2.16	1.67–2.80	**< 0.001**	2.15	1.66–2.79	**< 0.001**	2.07	1.08–3.98	**0.030**	1.89	0.94–3.76	0.072
Quartile 4	2.34	1.82–3.00	**< 0.001**	2.29	1.77–2.95	**< 0.001**	2.01	1.02–3.97	**0.045**	2.08	1.03–4.20	**0.040**
**TyG-BMI categories**												
Quartile 1	1	—	—	1	—	—	1	—	—	1	—	—
Quartile 2	1.48	1.13–1.95	**0.005**	1.52	1.15–2.01	**0.003**	1.90	1.07–3.38	**0.030**	1.99	1.09–3.63	**0.026**
Quartile 3	2.23	1.72–2.89	**< 0.001**	2.22	1.71–2.90	**< 0.001**	1.89	1.01–3.52	**0.045**	1.93	1.00–3.70	**0.050**
Quartile 4	2.96	2.29–3.82	**< 0.001**	2.93	2.26–3.80	**< 0.001**	2.83	1.52–5.27	**0.001**	2.80	1.45–5.39	**0.002**
**TyG-WC categories**												
Quartile 1	1	—	—	1	—	—	1	—	—	1	—	—
Quartile 2	1.91	1.46–2.49	**< 0.001**	1.86	1.42–2.44	**< 0.001**	1.75	0.89–3.45	0.104	1.87–	0.90–3.57	0.098
Quartile 3	2.33	1.71–2.90	**< 0.001**	2.18	2.67–2.84	**< 0.001**	3.04	1.54–5.97	**0.001**	3.18	1.55–6.51	**0.002**
Quartile 4	3.23	2.49–4.19	**< 0.001**	3.09	2.38–4.02	**< 0.001**	2.33	1.21–4.47	**0.011**	2.49	1.26–4.91	**0.009**

Model 1: adjusted for sex in 2009.

Model 2: model 1+ smoke habits, alcohol consumption, community type, married status and education years.

HOMA-IR, homeostasis model assessment of insulin resistance; HOMA-β, homeostasis model assessment of β-cell function; TyG, triglyceride and glucose; VAI, visceral adiposity index; LAP, lipid accumulation product; BMI, body mass index.

Significant values were in bold.

### Sensitivity analysis

Further analysis was conducted to explore whether lipid-based IR substitution indices could predict hypertension independent of IR. Supplementary Table 3 further adjusted the HOMA-IR and showed a similar trend in the significant prediction of hypertension by the substitute lipid-based IR indices. The sex-specific and age-specific relationship between the lipid-based IR index and the development of hypertension independent of IR is shown in Supplementary Tables 4, 5, respectively. The TyG-BMI and TyG-WC still showed distinctly higher contributions to hypertension in females than in males. A similar trend exists in the predictive role of hypertension by lipid-based index in the comparison between young and elderly individuals before and after the adjustment for IR.

Supplementary Table 6 shows the areas under the ROC curve (AUROCs), optimal cut-off values, sensitivities, and specificities for IR indices associated with hypertension. The TyG-WC showed the highest AUROC value for predicting hypertension (0.618, 95% CI 0.597–0.639). The TyG-WC cut-off value for predicting hypertension in the whole group was 635.88, with 79.0% sensitivity and 39.1% specificity, which provided extra evidence for the superiority of the TyG-BMI and TyG-WC compared with the HOMA-IR in clinical hypertension prediction. Furthermore, we calculated the cut-off value for predicting hypertension according to different obese statuses (Supplementary Table 7) and found that the cut-off value of TyG-BMI for predicting hypertension was 187.86, with 41.8% sensitivity and 70.7% specificity in the subjects with normal BMI, and was 233.33 with 34.7% sensitivity and 71.1% specificity in the overweight subjects.

## Discussion

With this large-scale representative longitudinal, community-based survey, we were the first to compare the six novel IR-related lipid indices and the HOMA-IR with incident hypertension. We confirmed the clinical usefulness of the surrogate IR index in the development of hypertension by sex and different age groups. Models combining the TyG with BMI and WC had a superior ability to predict the presence of hypertension, as did most of the surrogate IR indices and the HOMA-IR. The ability to predict hypertension by the IR index was most notable in young individuals (18 ≤ age ≤ 64 years), which still existed independent of the HOMA-IR level.

Compelling evidence has demonstrated that IR promotes the development of hypertension and CVDs (35–37). The HOMA-IR is well-recognized as a reliable marker to evaluate IR and could be defined as an IR outcome (14). In this study, subjects in quartile 4 had significantly higher risks of hypertension than those in quartile 1 after being adjusted by sex and age, smoke habits, alcohol consumption, community type, married status, and education years. The mechanism of the relationship between IR and the development of hypertension has not been fully elucidated. A previous study looked at renal sodium retention and the subsequent renin-angiotensin aldosterone system (RAAS) (38). Endothelial dysfunction, sympathetic nervous system activity, and vascular resistance caused by IR may play crucial roles in the pathogenesis of the development of increased blood pressure (39, 40). Additionally, IR was reported to be induced through chronic low-grade inflammation by adipocytokines (12), which may be related to hypertension. The HOMA-β used to assess the insulin secretory capacity of pancreatic beta cells was not significantly associated with the presence of hypertension in this study, indicating that the interpretation and extrapolation of the HOMA-β value in the application of predicting hypertension should be performed cautiously.

Six surrogate IR indices related to lipid markers in this study were found to be associated with advanced BP. This is advantageous for clinical studies, as it is a routine test performed in primary care settings and a simple and inexpensive parameter to assess adiposity, hyperlipidemia, and other CV risks. A previous cross-sectional epidemiological study of the Romanian population showed the association between the presence of hypertension and six surrogate IR indices (22). Recent studies demonstrated that the TyG, TyG-BMI, and TyG-WC indicators can comprehensively assess TG, FPG, visceral fat, and IR, as a correlation exists between these indices and other IR indices, such as the HOMA-IR (41). The combination of the TyG and adiposity evaluation *via* BMI and WC was reported to increase the strength of early IR diagnosis in Asian patients (15, 42). In this study, we found the superiority of the TyG-BMI and TyG-WC compared with the HOMA-IR in the prediction of hypertension. The better ability of the TyG-BMI and the TyG-WC index to predict incident hypertension compared with the HOMA-IR is of clinical relevance and may be possibly explained by the fact that they take body fat distribution into consideration. VAI and LAP, indicators of visceral adiposity, showed a strong association with advanced hypertension in the current study. It has been acknowledged that visceral obesity is more important in modulating IR because of the lipotoxicity caused by ectopic lipid deposits (43). Similar results were seen in the relationship between TG/HDL-C and hypertension.

The differences in the relationship between the surrogate IR index and subsequent hypertension by sex were shown in the current study. The relationship between IR and BP has been reported to be sex-specific (44, 45). Regarding the non-insulin-based index reflecting IR, the TyG-BMI and TyG-WC performed better in predicting hypertension in females than in males in the current study. A recent study based on the CHNS (2009–2011) indicated that VAI scores were significantly and positively associated with BP levels and the prevalence of hypertension in males after full adjustment (46), which was contrary to our findings. With regard to the relationship between the IR index and the presence of hypertension by sex, HOMA-IR, VAI, LAP, TyG-BMI, and TyG-WC in females showed higher predictivity in hypertension than in males. A recent study reported a higher prevalence of IR based on the HOMA-IR in hypertensive males than in normotensive males, which did not exist in females in a study involving African-American participants (47), and this finding was contrary to our sex-specific HOMA-IR findings. Our findings seem to indicate that obesity, either general or central, or subcutaneous and visceral fat accumulation is a more prominent risk factor for hypertension among females than males. Age and race differences in the study population, the statistical methods, and the measurement of IR may all have contributed to this gender discrepancy. More, well-designed cohort studies are required to further investigate the sex-specific relationship between IR and hypertension. The predictive role independent of IR by different surrogate IR indices in the presence of hypertension indicated that it was more likely related to lipid deposition.

The relationship between the IR index and hypertension was specific in different age groups in this study. The HOMA-IR had a stronger ability to predict incident hypertension for young participants (18 ≤ age ≤ 64 years) than for elderly participants (age ≥ 65 years old). Similar results were found in the predictive role of the TyG, VAI, LAP, TyG-BMI, and TyG-WC in hypertension, suggesting that for young individuals, the combination of lipid and insulin parameters has the ability to predict hypertension. Our findings indicated more clinical value for the younger Asian population that may reduce the burden of subsequent CVDs caused by advanced BP later in life.

Our study has several strengths, including a well-established cohort of the Chinese population, diverse population-based designs, prospective follow-up designs, and detailed measurements of lipid, insulin, and glucose metabolism. We performed a comprehensive comparison of non-insulin surrogate IR indices and their insulin-based parameters to determine their ability to predict the presence of hypertension. However, several limitations of this study must be mentioned. First, we did not perform a hyperinsulinemic-euglycemic clamp, which is the gold standard to assess insulin sensitivity and insulin secretion. Racial homogeneity was also a limitation of this study. The results of this study cannot be generalized to other ethnic populations; hence, further research is needed in the field. Long-term follow-up cohort studies are needed to validate our findings.

## Conclusion

Our results provide evidence for the superiority of the TyG-BMI and TyG-WC compared with the HOMA-IR in the prediction of clinical hypertension through this large sample representative of our population, indicating the clinical value of comprehensively evaluating blood triglycerides, blood glucose, visceral fat, and IR. Prediction by the surrogate IR index in hypertension exists even after further adjustment for the HOMA-IR, indicating that the predictive value of lipid deposition in hypertension cannot be ignored. The sex-specific predictive value is distinct for different IR indicators. It is valuable to assess these IR indices to prevent the development of hypertension in young individuals, which is beneficial for the efficient allocation of public resources to medical therapy.

## Data availability statement

The datasets presented in this study can be found in online repositories. The names of the repository/repositories and accession number(s) can be found at: http://www.cpc.unc.edu/projects/china.

## Ethics statement

The CHNS study was approved by the Institutional Review Committees of the National Institute of Nutrition and Food Safety, the Chinese Center for Disease Control and Prevention, the University of North Carolina at Chapel Hill, and the China-Japan Friendship Hospital, Ministry of Health. The protocols were in accordance with relevant guidelines and regulations. The patients/participants provided their written informed consent to participate in this study.

## Author contributions

YY collected the data, performed the statistical analysis, and was charge of writing, drafting, and preparation of the manuscript. WS and XK were charge of conception or design, interpretation of data, and revision of the manuscript. All the authors approved the final version of the manuscript.

## Funding

This work was supported by the 70th batch of China Postdoctoral Science Foundation (2021M701762), Postdoctoral Research Program of Jiangsu Province (2021K077A), and Doctoral Program of Entrepreneurship and Innovation in Jiangsu Province (JSSCBS20211480). Many thanks to the National Institute for Health (NIH) Fogarty program (D43 TW009077) and the Eunice Kennedy Shriver National Institute of Child Health and Human Development (NICHD, R01 HD30880; P2C HD050924) for financial support for the CHNS data collection and analysis files from 1989 to 2015 and future surveys, the China-Japan Friendship Hospital, the Ministry of Health for support for CHNS 2009, the Chinese National Human Genome Center at Shanghai since 2009, and the Beijing Municipal Center for Disease Prevention and Control since 2015.

## Conflict of interest

The authors declare that the research was conducted in the absence of any commercial or financial relationships that could be construed as a potential conflict of interest.

## Publisher's note

All claims expressed in this article are solely those of the authors and do not necessarily represent those of their affiliated organizations, or those of the publisher, the editors and the reviewers. Any product that may be evaluated in this article, or claim that may be made by its manufacturer, is not guaranteed or endorsed by the publisher.

## Abbreviations

IR: insulin resistance
CHNS: China Health and Nutrition Survey
HOMA-IR: homeostasis model assessment of insulin resistance
TyG: triglyceride-glucose index
BMI: body mass index
WC: waist circumference
CVD: cardiovascular disease
TG: triglyceride
HDL-C: high-density lipoprotein cholesterol
FPG: fasting plasma glucose
VAI: visceral adiposity index
LAP: lipid accumulation product
BP: Blood pressure
SBP: systolic blood pressure
DBP: diastolic blood pressure
MAP: mean arterial pressure
ApoA: apolipoprotein A
ApoB: apolipoprotein B
TC: total cholesterol
LDL-C: low-density lipoprotein cholesterol
OR: odds ratio
CI: confidence intervals
RAAS: renin-angiotensin aldosterone system.
