# Supplementary material for: Comparison between distinct insulin resistance indices in measuring the development of hypertension: The China Health and Nutrition Survey
Source: Front Cardiovasc Med. 2022 Oct 6;9:912197. doi: 10.3389/fcvm.2022.912197 (PMC9582523; doi:10.3389/fcvm.2022.912197)
Supplement: Supplementary file 1 [file Table_1.docx]

| **Table S1. Comparison of waist circumference and its related product between target population with normal blood pressure and hypertension by gender** | | | |
| --- | --- | --- | --- |
| Parameter | Subjects with normal blood pressure | Subjects with hypertension | *P* value |
| Male |  |  |  |
| WC in 2009, cm | 81.1(74.5-88.4) | 85.0(78.0-91.0) | <0.001 |
| WC in 2015, cm | 84.0(77.0-91.0) | 88.0(81.0-95.0) | <0.001 |
| VAI | 1.09(0.69-2.06) | 1.28(0.73-2.08) | 0.010 |
| LAP | 19.19(8.26-36.97) | 25.26(12.90-43.51) | <0.001 |
| TyG-WC | 688.29(606.75-775.56) | 728.27(647.94-807.31) | <0.001 |
| Female |  |  |  |
| WC in 2009, cm | 78.6(72.0-85.0) | 83.0(77.0-90.5) | <0.001 |
| WC in 2015, cm | 81.0(74.1-87.0) | 85.4(79.0-92.1) | <0.001 |
| VAI | 1.43(0.88-2.36) | 1.65(1.05-2.69) | <0.001 |
| LAP | 22.60(12.40-39.69) | 31.02(18.55-54.37) | <0.001 |
| TyG-WC | 655.80(588.58-731.68) | 705.07(642.94-794.52) | <0.001 |
| Abbreviations: WC=waist circumference; VAI= visceral adiposity index; LAP= lipid accumulation product; TyG =triglyceride and glucose;.  Continuous variables are shown as median (quartile 1, quartile 3) if non-normally distributed. | | | |
