# Supplementary material for: Comparison between distinct insulin resistance indices in measuring the development of hypertension: The China Health and Nutrition Survey
Source: Front Cardiovasc Med. 2022 Oct 6;9:912197. doi: 10.3389/fcvm.2022.912197 (PMC9582523; doi:10.3389/fcvm.2022.912197)
Supplement: Supplementary file 2 [file Table_2.docx]

| **Table S2. Comparison of characteristics and cardiovascular risks between target population by different age periods** | | | |
| --- | --- | --- | --- |
| Parameter | Yong participants  (18≤ age≤64 years old) | Elderly participants  (age≥65 years old) | *P* value |
| Gender (%) |  |  | 0.082 |
| Male | 1243(42.5) | 189(47.4) |  |
| Parameter in 2009 |  |  |  |
| Age, yr | 46(38-54) | 69(67-73) |  |
| BMI, kg/m^2^ | 23.0±3.1 | 22.4±3.5 | <0.001 |
| WC, cm | 83(76-90) | 84(78-91) | 0.001 |
| Hip, cm | 95(90-100) | 93(88-98) | 0.084 |
| SBP, mm Hg | 118(109-122) | 121(114-130) | <0.001 |
| DBP, mm Hg | 78(70-81) | 78(70-81) | 0.656 |
| MAP, mm Hg | 90(84-95) | 92(87-97) | <0.001 |
| Urea, mmol/L | 5.33±1.46 | 5.99±1.56 | <0.001 |
| Serum uric acid, μmol/L | 279(229-341) | 302(243-360) | <0.001 |
| Serum creatinine, μmol/L | 83(75-93) | 90(80-101) | <0.001 |
| HDL-C, mmol/L | 1.46±0.45 | 1.50±0.39 | 0.013 |
| LDL-C, mmol/L | 2.91±0.89 | 3.10±1.06 | <0.001 |
| TC, mmol/L | 4.78±0.95 | 4.94±0.92 | 0.001 |
| Triglycerides, mmol/L | 1.58±1.35 | 1.45±1.08 | 0.391 |
| Insulin, IU/mL | 10.00(7.13-14.16) | 8.97(6.42-14.32) | 0.023 |
| Total protein, g/L | 77(74-80) | 77(74-80) | 0.624 |
| Albumin, g/L | 47(45-49) | 46(44-48) | <0.001 |
| Fasting plasma glucose, mmol/L | 5.23±1.22 | 5.55±1.33 | <0.001 |
| Alanine Aminotransferase, U/L | 18(13-26) | 17(13-23) | 0.001 |
| Apolipoprotein A, g/L | 110(95-129) | 114(100-130) | 0.020 |
| Apolipoprotein B, g/L | 85(70-104) | 91(74-108) | 0.001 |
| HOMA-IR | 2.23(1.56-3.30) | 2.12(1.44-3.46) | 0.433 |
| HOMA-β | 133.06(89.76-198.65) | 104.14(71.90-164.76) | <0.001 |
| TyG | 8.43(8.03-8.89) | 8.42(8.07-8.93) | 0.443 |
| TG/HDL-C | 0.84(0.52-1.41) | 0.79(0.50-1.28) | 0.130 |
| VAI | 1.35(0.83-2.32) | 1.30(0.77-2.27) | 0.280 |
| LAP | 22.87(11.85-42.00) | 23.04(12.82-43.00) | 0.498 |
| TyG-BMI | 192.62(169.73-218.87) | 187.49(163.50-215.47) | 0.010 |
| TyG-WC | 680.20(603.87-764.09) | 692.66(626.17-784.74) | 0.008 |
| Parameter in 2015 |  |  |  |
| SBP in 2015, mm Hg | 122(114-134) | 132(121-144) | <0.001 |
| DBP in 2015, mm Hg | 80(73-85) | 80(72-85) | 0.072 |
| MAP in 2015, mm Hg | 94(88-101) | 97(91-104) | <0.001 |
| BMI in 2015, kg/m^2^ | 23.6(21.5-25.9) | 22.6(20.3-25.4) | <0.001 |
| WC in 2015, cm | 83(76-90) | 84(78-91) | 0.039 |
| Hip in 2015, cm | 95(90-100) | 93(88-98) | 0.004 |
| Abbreviations: BMI=body mass index; WC=waist circumference; SBP=systolic blood pressure; DBP=diastolic blood pressure; MAP= mean arterial pressure; HDL-C= high-density lipoprotein cholesterol, LDL-C= low-density lipoprotein cholesterol; TC= total cholesterol; HOMA-IR= homeostasis model assessment of insulin resistance; HOMA-β= homeostasis model assessment of β-cell function; TyG =triglyceride and glucose; VAI= visceral adiposity index; LAP= lipid accumulation product.  Continuous variables are shown as mean ± SD if normally distributed or median (quartile 1, quartile 3) if non-normally distributed. Categorical variables are expressed as numbers and percentages of subjects.  Education year, BMI , urea, HDL-C, LDL-C, TC, triglycerides, white blood cell count, red blood cell count, platelet count, hemoglobin A1c, hemoglobin, fasting plasma glucose in 2009 were normally distributed. | | | |
