# Supplementary material for: Comparison between distinct insulin resistance indices in measuring the development of hypertension: The China Health and Nutrition Survey
Source: Front Cardiovasc Med. 2022 Oct 6;9:912197. doi: 10.3389/fcvm.2022.912197 (PMC9582523; doi:10.3389/fcvm.2022.912197)
Supplement: Supplementary file 3 [file Table_3.docx]

| **Table S3. Risk ratios and 95% confidence intervals of the association of lipid-based index with incident hypertension independent of IR** | | | | |
| --- | --- | --- | --- | --- |
| Categories | Model 1 | | Model 2 | |
|  | RR（95%CI） | *P* value | RR（95%CI） | *P* value |
| **TyG** **categories** |  |  |  |  |
| Quartile 1 | 1 | - | 1 | - |
| Quartile 2 | 1.31（1.03-1.67） | **0.030** | 1.14(0.88-1.49) | 0.325 |
| Quartile 3 | 1.71（1.34-2.17） | **<0.001** | 1.40(1.04-1.87) | **0.025** |
| Quartile 4 | 1.84（1.44-2.34） | **<0.001** | 1.50(1.04-2.17) | **0.030** |
| **TG/HDL-C categories** |  |  |  |  |
| Quartile 1 | 1 | - | 1 | - |
| Quartile 2 | 1.13（0.89-1.43） | 0.308 | 0.98(0.75-1.28) | 0.904 |
| Quartile 3 | 1.52（1.20-1.91） | **<0.001** | 1.14(0.85-1.53) | 0.388 |
| Quartile 4 | 2.16（1.16-1.86） | **0.001** | 0.91(0.62-1.33) | 0.629 |
| **VAI categories** |  |  |  |  |
| Quartile 1 | 1 | - | 1 | - |
| Quartile 2 | 1.29（1.02-1.64） | 0.033 | 1.18(0.90-1.54) | 0.229 |
| Quartile 3 | 1.69（1.33-2.15） | **<0.001** | 1.40(1.03-1.90) | **0.033** |
| Quartile 4 | 1.57（1.23-2.00） | **<0.001** | 1.06(0.74-1.52) | 0.752 |
| **LAP categories** |  |  |  |  |
| Quartile 1 | 1 | - | 1 | - |
| Quartile 2 | 1.52（1.19-1.96） | **0.001** | 1.39(1.06-1.81) | **0.016** |
| Quartile 3 | 2.03（1.58-2.59） | **<0.001** | 1.62(1.22-2.16) | **0.001** |
| Quartile 4 | 2.17（1.70-2.78） | **<0.001** | 1.88(1.33-2.64) | **<0.001** |
| **TyG-BMI categories** |  |  |  |  |
| Quartile 1 | 1 | - | 1 | - |
| Quartile 2 | 1.57（1.22-2.03） | **0.001** | 1.48(1.13-1.93) | **0.004** |
| Quartile 3 | 2.13（1.66-2.73） | **<0.001** | 1.94(1.46-2.58) | **<0.001** |
| Quartile 4 | 2.98（2.33-3.82） | **<0.001** | 2.72(1.99-3.73) | **<0.001** |
| **TyG-WC categories** |  |  |  |  |
| Quartile 1 | 1 | - | 1 | - |
| Quartile 2 | 1.76（1.36-2.27） | **<0.001** | 1.69(1.29-2.23) | **<0.001** |
| Quartile 3 | 2.14（1.65-2.76） | **<0.001** | 1.93(1.43-2.60) | **<0.001** |
| Quartile 4 | 3.00（2.33-3.86） | **<0.001** | 2.55(1.81-3.58) | **<0.001** |
| Model 1: adjusted for sex, age, smoke habits, alcohol consumption, community type, married status and education years, homeostasis model assessment of insulin resistance(HOMA-IR) ;  Model 2: model 1+ urea, serum uric acid, serum creatinine, low-density lipoprotein cholesterol, total cholesterol, white blood cell count, red blood cell count, platelet count, hemoglobin A1c, hemoglobin, total protein, albumin, alanine aminotransferase, apolipoprotein A, apolipoprotein B.  TyG =triglyceride and glucose; VAI= visceral adiposity index; LAP= lipid accumulation product; BMI= body mass index, WC=waist circumulence. | | | | |
