# Supplementary material for: Comparison between distinct insulin resistance indices in measuring the development of hypertension: The China Health and Nutrition Survey
Source: Front Cardiovasc Med. 2022 Oct 6;9:912197. doi: 10.3389/fcvm.2022.912197 (PMC9582523; doi:10.3389/fcvm.2022.912197)
Supplement: Supplementary file 4 [file Table_4.docx]

| **Table S4. Risk ratios and 95% confidence intervals of the association of lipid-based index with incident hypertension independent of HOMA-IR by sex** | | | | |
| --- | --- | --- | --- | --- |
| Categories | Men | | Women | |
|  | RR（95%CI） | *P* value | RR（95%CI） | *P* value |
| **TyG** **categories** |  |  |  |  |
| Quartile 1 | 1 | - | 1 | - |
| Quartile 2 | 1.24(0.86-1.78) | 0.259 | 1.26(0.89-1.76) | 0.189 |
| Quartile 3 | 1.83(1.29-2.61) | **0.001** | 1.42(1.01-2.00) | **0.042** |
| Quartile 4 | 1.90(1.33-2.70) | **<0.001** | 1.58(1.10-2.26) | **0.014** |
| **TG/HDL-C categories** |  |  |  |  |
| Quartile 1 | 1 | - | 1 | - |
| Quartile 2 | 0.92(0.64-1.34) | 0.665 | 1.24(0.91-1.70) | 0.178 |
| Quartile 3 | 1.54(1.09-2.19) | **0.016** | 1.37(0.99-1.89) | 0.058 |
| Quartile 4 | 1.46(1.03-2.07) | **0.036** | 1.27(0.91-1.78) | 0.166 |
| **VAI categories** |  |  |  |  |
| Quartile 1 | 1 | - | 1 | - |
| Quartile 2 | 1.04(0.75-1.44) | 0.822 | 1.54(1.07-2.21) | **0.021** |
| Quartile 3 | 1.72(1.23-2.39) | **0.001** | 1.52(1.05-2.19) | **0.025** |
| Quartile 4 | 1.33(0.94-1.88) | 0.112 | 1.61(1.11-2.31) | **0.011** |
| **LAP categories** |  |  |  |  |
| Quartile 1 | 1 | - | 1 | - |
| Quartile 2 | 1.56(1.11-2.18) | **0.011** | 1.46(1.00-2.13) | **0.050** |
| Quartile 3 | 1.92(1.37-2.69) | **<0.001** | 1.96(1.35-2.83) | **<0.001** |
| Quartile 4 | 1.89(1.34-2.67) | **<0.001** | 2.22(1.53-3.23) | **<0.001** |
| **TyG-BMI categories** |  |  |  |  |
| Quartile 1 | 1 | - | 1 | - |
| Quartile 2 | 1.41(0.97-2.03) | **0.070** | 1.67(1.17-2.41) | **0.005** |
| Quartile 3 | 2.02(1.42-2.88) | **<0.001** | 2.08(1.46-2.98) | **<0.001** |
| Quartile 4 | 2.77(1.93-3.99) | **<0.001** | 2.83(1.98-4.04) | **<0.001** |
| **TyG-WC categories** |  |  |  |  |
| Quartile 1 | 1 | - | 1 | - |
| Quartile 2 | 1.70(1.16-2.51) | **0.007** | 1.69(1.19-2.38) | **0.003** |
| Quartile 3 | 1.82(1.24-2.68) | **0.002** | 2.24(1.57-3.19) | **<0.001** |
| Quartile 4 | 2.72(1.88-3.94) | **<0.001** | 2.88(1.99-4.17) | **<0.001** |
| Adjusted model : adjusted for age, smoke habits, alcohol consumption, community type, married status and education years, homeostasis model assessment of insulin resistance(HOMA-IR) ;  TyG =triglyceride and glucose; VAI= visceral adiposity index; LAP= lipid accumulation product; BMI= body mass index, WC=waist circumulence. | | | | |
