# Supplementary material for: Comparison between distinct insulin resistance indices in measuring the development of hypertension: The China Health and Nutrition Survey
Source: Front Cardiovasc Med. 2022 Oct 6;9:912197. doi: 10.3389/fcvm.2022.912197 (PMC9582523; doi:10.3389/fcvm.2022.912197)
Supplement: Supplementary file 5 [file Table_5.docx]

| **Table S5. Risk ratios and 95% confidence intervals of the association of lipid-based index with incident hypertension independent of HOMA-IR by different age periods** | | | | |
| --- | --- | --- | --- | --- |
| Categories | Yong participants  (18≤ age≤64 years old) | | Elderly participants  (age≥65 years old) | |
|  | RR（95%CI） | *P* value | RR（95%CI） | *P* value |
| **TyG** **categories** |  |  |  |  |
| Quartile 1 | 1 | - | 1 | - |
| Quartile 2 | 1.37(1.06-1.77) | **0.018** | 1.12(0.59-2.14) | 0.734 |
| Quartile 3 | 1.81(1.41-2.34) | **<0.001** | 1.63(0.80-3.34) | 0.183 |
| Quartile 4 | 1.90(1.47-2.45) | **<0.001** | 3.00(1.41-6.37) | **0.004** |
| **TG/HDL-C categories** |  |  |  |  |
| Quartile 1 | 1 | - | 1 | - |
| Quartile 2 | 1.19(0.92-1.53) | 0.188 | 0.93(0.51-1.70) | 0.820 |
| Quartile 3 | 1.56(1.22-1.99) | **<0.001** | 1.18(1.18-2.19) | 0.592 |
| Quartile 4 | 1.50(1.16-1.93) | **0.002** | 1.28(0.66-2.47) | 0.464 |
| **VAI categories** |  |  |  |  |
| Quartile 1 | 1 | - | 1 | - |
| Quartile 2 | 1.41(1.09-1.82) | **0.008** | 0.82(0.44-1.56) | 0.548 |
| Quartile 3 | 1.83(1.41-2.37) | **<0.001** | 1.25(0.67-2.36) | 0.487 |
| Quartile 4 | 1.68(1.30-2.18) | **<0.001** | 1.11(0.56-2.17) | 0.770 |
| **LAP categories** |  |  |  |  |
| Quartile 1 | 1 | - | 1 | - |
| Quartile 2 | 1.55(1.19-2.03) | **0.001** | 2.30(1.21-4.37) | **0.011** |
| Quartile 3 | 2.16(1.66-2.80) | **<0.001** | 1.91(0.96-3.82) | 0.067 |
| Quartile 4 | 2.33(1.79-3.02) | **<0.001** | 2.13(1.05-4.34) | **0.037** |
| **TyG-BMI categories** |  |  |  |  |
| Quartile 1 | 1 | - | 1 | - |
| Quartile 2 | 1.52(1.15-2.01) | **0.003** | 2.05(1.12-3.77) | **0.021** |
| Quartile 3 | 2.23(1.71-2.90) | **<0.001** | 1.88(0.96-3.67) | 0.066 |
| Quartile 4 | 3.06(2.35-4.00) | **<0.001** | 3.15(1.59-6.24) | **0.001** |
| **TyG-WC categories** |  |  |  |  |
| Quartile 1 | 1 | - | 1 | - |
| Quartile 2 | 1.88(1.43-2.47) | **<0.001** | 1.75(0.87-3.49) | 0.114 |
| Quartile 3 | 2.21(1.69-2.89) | **<0.001** | 3.51(1.68-7.32) | **0.001** |
| Quartile 4 | 3.20(2.44-4.20) | **<0.001** | 2.78(1.37-5.64) | **0.005** |
| Adjusted model : adjusted for sex, smoke habits, alcohol consumption, community type, married status and education years, homeostasis model assessment of insulin resistance(HOMA-IR) ;  TyG =triglyceride and glucose; VAI= visceral adiposity index; LAP= lipid accumulation product; BMI= body mass index, WC=waist circumulence. | | | | |
