# Supplementary material for: Comparison between distinct insulin resistance indices in measuring the development of hypertension: The China Health and Nutrition Survey
Source: Front Cardiovasc Med. 2022 Oct 6;9:912197. doi: 10.3389/fcvm.2022.912197 (PMC9582523; doi:10.3389/fcvm.2022.912197)
Supplement: Supplementary file 6 [file Table_6.docx]

| Table S6. The areas under the ROC curve (AUROCs), optimal cut-off values, sensitivities and specificities for parameters associated with hypertension | | | | | |
| --- | --- | --- | --- | --- | --- |
| Parameters | AUROC (95% CI) | Cut-off point | Sensitivity (%) | Specificity (%) | P value |
| HOMA-IR | 0.538(0.516-0.560) | 3.128 | 33.3 | 74.1 | 0.001 |
| HOMA-β | 0.458(0.435-0.480) | 328.313 | 8.3 | 92.3 | <0.001 |
| TyG | 0.572(0.550-0.594) | 8.42 | 59.3 | 52.2 | <0.001 |
| TG/HDL-C | 0.548(0.526-0.570) | 0.84 | 55.6 | 53.3 | <0.001 |
| VAI | 0.546(0.524-0.568) | 1.10 | 66.0 | 42.0 | <0.001 |
| LAP | 0.591(0.569-0.612) | 18.120 | 71.1 | 43.8 | <0.001 |
| TyG-BMI | 0.609(0.588-0.631) | 194.817 | 60.2 | 58.1 | <0.001 |
| TyG-WC | 0.618(0.597-0.639) | 635.877 | 79.0 | 39.1 | <0.001 |
| BMI | 0.606(0.584-0.627) | 23.826 | 49.6 | 67.6 | <0.001 |
| TC | 0.570(0.548-0.592) | 4.885 | 50.8 | 60.6 | <0.001 |
| TG | 0.557(0.535-0.578) | 1.335 | 49.2 | 61.1 | <0.001 |
| LDL-C | 0.566(0.544-0.588) | 3.095 | 47.7 | 63.2 | <0.001 |
| insulin | 0.518(0.496-0.541) | 9.87 | 53.1 | 51.1 | 0.111 |
| Uric acid | 0.554(0.532-0.576) | 285.5 | 54.8 | 54.3 | <0.001 |
| glucose | 0.580(0.558-0.602) | 4.983 | 64.2 | 48.2 | <0.001 |
| creatinine | 0.535(0.513-0.558) | 82.5 | 57.9 | 49.0 | 0.002 |
| HOMA-IR= homeostasis model assessment of insulin resistance; HOMA-β= homeostasis model assessment of β-cell function; TyG =triglyceride and glucose; VAI= visceral adiposity index; LAP= lipid accumulation product; BMI=body mass index; TC= total cholesterol; TG=triglycerides; LDL-C= low-density lipoprotein cholesterol; | | | | | |
