# Supplementary material for: Comparison between distinct insulin resistance indices in measuring the development of hypertension: The China Health and Nutrition Survey
Source: Front Cardiovasc Med. 2022 Oct 6;9:912197. doi: 10.3389/fcvm.2022.912197 (PMC9582523; doi:10.3389/fcvm.2022.912197)
Supplement: Supplementary file 7 [file Table_7.docx]

| Table S7. The areas under the ROC curve (AUROCs), optimal cut-off values, sensitivities and specificities for LAP associated with hypertension according to adiposity status | | | | | |
| --- | --- | --- | --- | --- | --- |
| Parameters | AUROC (95% CI) | Cut-off point | Sensitivity (%) | Specificity (%) | P value |
| Subjects with normal weight | | | | | |
| HOMA-IR | 0.521(0.491-0.551) | 3.13 | 25.5 | 80.9 | 0.163 |
| TyG | 0.579(0.549-0.608) | 8.42 | 51.4 | 61.1 | **<0.001** |
| TG/HDL-C | 0.551(0.522-0.581) | 0.84 | 46.5 | 63.2 | **0.001** |
| VAI | 0.546(0.516-0.576) | 1.04 | 60.8 | 47.9 | **0.002** |
| LAP | 0.577(0.548-0.607) | 17.69 | 55.9 | 57.5 | **<0.001** |
| TyG-BMI | 0.581(0.551-0.610) | 187.86 | 41.8 | 70.7 | **<0.001** |
| TyG-WC | 0.604(0.576-0.633) | 628.12 | 68.5 | 50.2 | **<0.001** |
| TC | 0.572(0.543-0.602) | 4.69 | 57.0 | 55.5 | **<0.001** |
| TG | 0.565(0.535-0.594) | 1.23 | 48.2 | 63.4 | **<0.001** |
| LDL-C | 0.558(0.528-0.588) | 2.73 | 61.2 | 49.3 | **<0.001** |
| insulin | 0.495(0.465-0.525) | 11.47 | 33.0 | 69.2 | 0.746 |
| Uric acid | 0.552(0.523-0.581) | 295.50 | 45.4 | 62.5 | **0.001** |
| glucose | 0.582(0.553-0.612) | 4.97 | 60.2 | 52.7 | **<0.001** |
| creatinine | 0.546(0.517-0.576) | 79.5 | 68.5 | 40.7 | **0.002** |
| Overweight subjects | | | | | |
| HOMA-IR | 0.510(0.471-0.549) | 3.61 | 30.6 | 73.4 | 0.620 |
| TyG | 0.504(0.465-0.542) | 8.74 | 48.8 | 55.3 | 0.859 |
| TG/HDL-C | 0.488(0.449-0.528) | 0.42 | 93.8 | 7.4 | 0.555 |
| VAI | 0.484(0.445-0.524) | 0.76 | 91.3 | 10.9 | 0.427 |
| LAP | 0.513(0.474-0.552) | 85.23 | 17.2 | 86.8 | 0.517 |
| TyG-BMI | 0.526(0.487-0.565) | 233.33 | 34.7 | 71.1 | 0.188 |
| TyG-WC | 0.549(0.510-0.588) | 749.38 | 62.2 | 48.2 | **0.014** |
| TC | 0.540(0.501-0.579) | 5.35 | 36.3 | 71.1 | **0.046** |
| TG | 0.492(0.453-0.531) | 0.63 | 96.6 | 6.9 | 0.678 |
| LDL-C | 0.549(0.510-0.588) | 3.70 | 26.9 | 81.4 | **0.014** |
| insulin | 0.508(0.468-0.547) | 16.35 | 26.6 | 77.5 | 0.701 |
| Uric acid | 0.551(0.513-0.590) | 259.50 | 75.0 | 37.0 | **0.010** |
| glucose | 0.530(0.491-0.569) | 5.04 | 64.4 | 42.6 | 0.135 |
| creatinine | 0.528(0.489-0.567) | 83.5 | 55.3 | 52.5 | 0.161 |
| Obese subjects |  |  |  |  |  |
| HOMA-IR | 0.429(0.352-0.506) | 0.97 | 100.0 | 2.3 | 0.073 |
| TyG | 0.450(0.373-0.527) | 8.24 | 85.2 | 16.7 | 0.207 |
| TG/HDL-C | 0.404(0.328-0.479) | 0.23 | 100 | 0.8 | **0.016** |
| VAI | 0.407(0.331-0.483) | 1.26 | 80.7 | 22.0 | **0.019** |
| LAP | 0.445(0.368-0.523) | 68.37 | 43.2 | 57.6 | 0.171 |
| TyG-BMI | 0.454(0.376-0.533) | 233.61 | 98.9 | 4.5 | 0.253 |
| TyG-WC | 0.506(0.427-0.584) | 899.71 | 38.6 | 609.7 | 0.890 |
| TC | 0.551(0.472-0.630) | 5.15 | 55.7 | 61.4 | 0.201 |
| TG | 0.421(0.345-0.497) | 0.93 | 86.4 | 14.4 | **0.047** |
| LDL-C | 0.578(0.502-0.654) | 2.91 | 69.3 | 49.2 | **0.049** |
| insulin | 0.404(0.328-0.481) | 3.92 | 100.0 | 2.3 | **0.007** |
| Uric acid | 0.572(0.543-0.601) | 182 | 98.9 | 2.3 | **0.016** |
| glucose | 0.565(0.488-0.642) | 5.50 | 52.3 | 61.4 | 0.102 |
| creatinine | 0.491(0.413-0.569) | 79.5 | 64.8 | 42.4 | 0.821 |
| HOMA-IR= homeostasis model assessment of insulin resistance; TyG =triglyceride and glucose; VAI= visceral adiposity index; LAP= lipid accumulation product; BMI=body mass index; TC= total cholesterol; TG=triglycerides; LDL-C= low-density lipoprotein cholesterol; | | | | | |
